# Impact of chronic consumption of probiotics, oats, and apples on expression of genes related to bile acids, lipid, gut peptides, and inflammation in peripheral monocular cells - findings from the CABALA study

**DOI:** 10.1007/s00394-025-03694-x

**Published:** 2025-05-10

**Authors:** Shouq Alzoufairi, Rose-Anna G. Pushpass, L. Liu, J. A. Lovegrove, K. G. Jackson

**Affiliations:** Hugh Sinclair Unit of Human Nutrition, Department of Food and Nutritional Sciences, Institute for Cardiovascular and Metabolic Research, Institute for Food, Nutrition and Health, University of Reading, Harry Nursten Building, Reading, RG6 6DZ UK

**Keywords:** Probiotics, Oats, Apples, Inflammation, Gene expression

## Abstract

**Purpose:**

Chronic intakes of functional foods (probiotics, apples and oats) have been reported to have beneficial effects on hepatic lipid regulation and glycaemic control, but mechanistic human studies humans are limited. An ex-vivo study was performed to determine the chronic effects of probiotics, oats, and apples on the expression of genes related to markers of cardiometabolic health in peripheral blood monocular cells (PBMC).

**Methods:**

In this CABALA sub-study (*n* = 59/61, age: 52 ± 12y), blood PBMC were also isolated before and 8 weeks after the daily consumption of either a probiotic with bile salt hydrolase activity *(Lactobacillus reuteri*), porridge oats, Renetta Canada apples or a control. Relative PBMC mRNA gene expression was determined and correlations performed between the fold change in response to the functional interventions and change in cardiometabolic disease risk markers.

**Results:**

Relative to baseline, there was an upregulation in the PBMC TLR4 mRNA expression in the control compared with the probiotics and apples groups (p$\:\le\:$0.024). Moderate inverse correlations were found between the fold change in GPBAR1 mRNA expression and change in plasma total and secondary BAs, HMGCR and SREBF1 mRNA gene expressions and high-density lipoprotein-cholesterol, and SREBF1 and GIPR mRNA gene expressions and glucose. TLR4 and TNFSF14 mRNA gene expressions were associated with pro-inflammatory cytokines (*p*=0.05).

**Conclusion:**

Probiotic and apples interventions attenuated the upregulation in PBMC TLR4 mRNA expression observed with the control. Correlations between fold change in mRNA gene expression and changes in cardiometabolic disease risk markers in response to the functional interventions were in agreement with previous studies.

**Clinical trial registry:**

The study was registered at clinical trials.gov (ref. NCT03369548).

## Introduction

In recent years, there has been a significant research focus on the role of human gut microbiota in maintaining host cardiometabolic health [1]. This has been driven by the findings from studies using functional interventions such as probiotics with bile salt hydrolase (BSH) activity, porridge oats high in fibre and β-glucan and polyphenol-rich foods which have reported beneficial effects on low-density lipoprotein-cholesterol (LDL-C) concentrations [2]. Changes in bile acid (BA) metabolism via modulation of the gut microbiota composition by these functional interventions has been proposed as a mechanism to explain these cholesterol-lowering effects [3, 4]. In the liver, BAs are synthesised from cholesterol by cholesterol 27 alpha-hydroxylase (CYP27A1) and released into the small intestine following food consumption to emulsify the dietary fats and to facilitate the intestinal absorption of lipids [5–8]. Within the gut, increased transformation of primary BA to secondary and tertiary BA by the microbiota, has been suggested to result in a net loss of cholesterol in the faeces and lead to a reduction in the recycling of primary BA by the enterohepatic circulation. As a result, hepatic intracellular cholesterol stores are lowered (8) promoting an upregulation in LDL-receptor (*LDL-R*) expression and clearance of circulating LDL. Both animal and human studies have demonstrated that BAs are signalling molecules that stimulate nuclear farnesoid X receptor (FXR), membrane G protein-coupled bile acid receptor-1 (GPBAR-1, also named Takeda G protein-coupled receptor 5 (TGR5)) and incretin hormones to regulate not only BA synthesis in the liver but also lipid, glucose, and energy metabolism [9].

Peripheral blood mononuclear cells (PBMC) are an easily accessible blood cell fraction primarily made up of lymphocytes and monocytes which are increasingly used as a biomarker tissue in nutrition studies. Most of the human genome is expressed by these cells, and they can reflect gene expression profile of internal tissues such as the liver in response to nutritional interventions [10]. Studies by ourselves and others have shown genes involved in hepatic cholesterol regulation to be responsive to dietary fat intake, and related to circulating LDL-C concentration [11, 12]. However, very little is known about how functional interventions impact on PBMC gene expression and how they relate to changes in circulating cardiometabolic disease risk markers. In the CirculAting Bile Acids as Biomarkers of Metabolic Health - Linking microbiotA (CABALA) Diet and Health study, we have previously reported chronic intake of the probiotic *Lactobacillus reuteri* with BSH activity to modulate postprandial plasma BA profiles whereas polyphenol-rich Renetta Canada apples and oats had favourable effects on postprandial glycaemia compared with the control [13]. Within the framework of the CABALA study, the aim of this secondary explorative analysis was to determine the expression of genes in PBMC related to the regulation of circulating BAs, lipids, glycaemia, gut peptides, and inflammatory markers after consuming the probiotic, oats, and apples interventions compared with the control. We hypothesised that consuming any of the functional interventions (probiotic, or oats, and apples) would have beneficial effects on mRNA gene expression and be related to favourable changes in circulating BAs and cardiometabolic disease (CMD) risk markers.

## Methods

### Study participants and design

A subset of fifty-nine healthy participants (aged between 18 and 70 years, BMI 20–32 kg/m^2^, and total cholesterol (TC) < 7.5 mmol/L) from the CABALA study (NCT03369548) were included in this secondary explorative analysis. Details of the study design, participant group, interventions, inclusion and exclusion criteria have been previously published [13]. The study was given a favourable opinion for conduct by the University of Reading Research Ethics Committee (Reference: 17/47). In brief, the participants followed one of the four different food interventions daily for 8 weeks: (i) 40 g/day of cornflakes (Kellogg’s, Michigan, USA) with two NCIMB 30242 probiotic capsules [UAS Labs Madison, WI, USA, > 2.5 billion CFU of *L. reuteri* per capsule (contained 28% (wt/wt) of *L. reuteri*, 69% (wt/wt) microcrystalline cellulose, 2% (wt/wt) magnesium stearate, and 1% (wt/wt) silica; Lot# TBMR 628-1] (probiotics group), (ii) 40 g/day of porridge oats (Quaker, Pepsico, UK) with two placebo capsules (UAS Labs Madison, WI, USA) (oats group), (iii) two Renetta Canada apples/day with two placebo capsules (Melinda SCA, Cles, Trentino) (apples group), or (iv) 40 g/day of cornflakes with two placebo capsules (control group). *L. reuteri*, a BSH-active strain, was the selected probiotic due to the reported hypocholesterolaemic (Total cholesterol and LDL-C) effects [14].

At baseline (week 0) and after the intervention period (week 8), anthropometrics and blood pressure were measured. A fasting blood sample was also collected for the isolation of PBMC and determination of plasma BAs (total BAs, conjugated BAs, unconjugated BAs, primary BAs, secondary BAs, hydrophobic BAs, and hydrophilic BAs), lipid profile (TC, LDL-C, high-density lipoprotein-cholesterol (HDL-C), triacylglycerol (TAG), and apolipoprotein B (apoB)), glucose, gut peptides (insulin and glucose dependent insulinotropic polypeptide (GIP)), inflammatory markers (interleukin (IL)-6, IL-18, tumour necrosis factor-alpha (TNF-α), and C-reactive protein (CRP)). Dietary intake was assessed using a 4-day weighed food diary on three weekdays and one weekend day prior to attending each study visit.

### PBMC isolation, cDNA synthesis and gene expression

PBMCs were isolated from the fasting blood samples by using the BD Vacutainer cell preparation tubes (BD, Biosciences, UK), as previously described [15]. Briefly, the blood was centrifuged to at 300 rpm (1700 x g) for 20 min at room temperature to isolate the white blood cell layer. The cells were washed twice by adding Dulbecco’s PBS without calcium and magnesium (Merck Life Science UK Ltd, UK) and centrifuging at room temperature at 1200 rpm (280 x g) for 20 min and 15 min respectively before the cell pellet was lysed with RLT buffer (Qiagen, UK) containing 1% Mercaptoethanol and then stored at -80^o^C. Total RNA was isolated using RNeasy Mini Kit (Qiagen Ltd.) according to the manufacturer’s instructions, RNA quantity and quality measured using the Nanodrop 1000 spectrophotometer (Nanodrop ND-1000 Thermo Fisher Scientific) before a SuperScript IV VILO Mastermix (Thermo Fisher Scientific, Winsford, UK) was used to synthesise cDNA from 1.2 µg total RNA. Samples were incubated at 25^o^C for 10 min (reaction volume = 10 µl) followed by 50^o^C for 10 min and 85^o^C for 5 min. UltraPure RNAse/DNAse free distilled water (Invitrogen) was used to dilute samples 1:10 before the cDNA concentration was quantified using a Nanodrop 1000 spectrophotometer and then stored at -20^o^C.

Prior to the start of the gene expression analysis, a human geNorm reference gene selection kit (Primerdesign Ltd, Camberley, UK) and qbase + software was used according to manufacturer’s protocol to screen 12 housekeeping genes in a representative subset of cDNA samples from the CABALA study [15]. Expression of the reference [beta-2-microglobulin (*B2M*) and beta-actin (*ACTB*)] and target genes was determined using 5ng/µl of cDNA by using real-time RT-PCR (QuantStudio3, Thermo Fisher Scientific) with TaqMan gene expression assays (Applied Biosystems) and normal cycling parameters. The target genes measured included *GPBAR1*, *CYP27A1*, nuclear receptor subfamily 1 group I member 2 (*NR1I2*), *LDL-R*, 3-hydroxy-3-methylglutaryl coenzyme A reductase (*HMGCR*), sterol regulatory element binding transcription factor 1 (*SREBF1*), peroxisome proliferator activated receptor d (*PPARD*), nuclear receptor subfamily 1 group H member 3 (*NR1H3*), acetyl-CoA acetyltransferase 1 (*ACAT1*), glucose-dependent insulinotropic polypeptide receptor (*GIPR*), toll-like receptor 4 (*TLR4*), tumour necrosis factor (ligand) superfamily member 14 (*TNFSF14*), and glucagon-like peptide 1 receptor (*GLP1R*). *NR1I2* and *GLP1R* were excluded from the data analysis due to the very low expression in the PBMC. Each target gene was normalised to the average of the two housekeeping genes before the fold change in mRNA expression relative to the baseline visit for each intervention was calculated by using the ΔΔCt method [16].

### Clinical and biochemical measurements

The methods for the collection of the fasting blood samples and analysis of the circulating BAs and cardiometabolic disease risk markers have been published in detail elsewhere [13]. Briefly, for the measurement of BAs, 50 µL of plasma, internal standard solution made up of known amounts of unconjugated (Spectra 2000 s.r.l., Italy), glycine conjugated (Cabru s.r.l., Italy), and taurine conjugated BAs (Sigma-Aldrich, Milan, Italy) were pipetted into an Ostro 96 well plate (Waters SPA, Italy), and filtration was performed using a positive pressure-96 manifold (Waters, USA). Ultra-HPLC (UHPLC) UltiMate 3000 (Dionex) coupled with a Triple Quad 5500 (AB Sciex) mass spectrometer (injected volume from prepared sample, 5 µL was used for the chromatographic separation of the compounds. BAs were classified according to their type and characteristics with 7 classes being used for statistical analysis; total, conjugated, unconjugated, primary, secondary, hydrophobic, and hydrophilic (the table of BA classification has been published [13]).

A Daytona plus clinical chemistry analyser (Randox Laboratories, County Antrim, UK), using kits supplied by Randox Laboratories was used to determine serum lipid concentrations (TC, HDL-C, and TAG), CRP and glucose. The Friedewald formula was applied to estimate LDL-C [17]. A Milliplex human metabolic hormone magnetic bead panel kit (Millipore Corporation, Billerica, MA, USA) determined plasma concentrations of GIP by using the Luminex 200 with xPONENT software version 4.2 (Invitrogen, Thermo Fisher Scientific). ELISA kits were used to measure insulin ((Crystal Chem, Elk Grove Village, IL, USA), and IL-18, with high-sensitivity assays used for TNF-α and IL-6 (Quantikine kits (Bio-Techne, Minneapolis, MN, USA) following the manufacturer’s instructions.

### Statistics analysis

As no formal sample size calculation was performed, the analysis performed in this manuscript is explorative, investigating the chronic effects of probiotic, oats, and apples interventions on the expression of genes related to BA and lipid metabolism, and inflammation in PBMC compared with the control. Changes in relative mRNA gene expression in the functional intervention groups combined will be related to the secondary outcomes of the CABALA study, which included changes in fasting circulating plasma BAs (total, conjugated, unconjugated, primary, secondary, hydrophobic, and hydrophilic BAs), fasting lipid profile (TC, HDL-C, TAG, LDL-C and apoB), glucose, gut peptides (insulin and GIP), and inflammatory markers (IL-6, IL-18, CRP, and TNF-a). The CABALA study was powered based on the change in postprandial BAs after fibre-rich baker products, as previously described [13].

Statistical analyses for baseline and changes in fasting cardiometabolic outcomes were completed using PROC MIXED methods using the SAS 9.4 statistical software (SAS Institute Inc.). Baseline and changes in fasting outcomes were formed on a logarithmic scale, adjusted for the age, sex and BMI. Data were back-transformed to the original scale for presentation in the tables. At baseline, subject characteristics between groups were examined with an F-Test, using post model fitting manipulation of results. The change in concentration of fasting measures from baseline to week 8 was analyzed using logarithmically transformed data (log week 8 concentration– log week 0 concentration = Δ (change from week 0)) before being statistically modelled. Lastly, participant was fitted as a random effect [18]. If there was a significant effect, post-hoc analysis was performed on the different intervention arms compared with the control group only.

IBM SPSS Statistics version 27 (SPSS Incl., IL, USA) was used for the gene expression statistical analyses. Normal distribution was assessed by the Shapiro-Wilk test. The relative fold changes in *ACAT1* and *GPBAR1* mRNA gene expression were not normally distributed, thus the non-parametric independent test was used for these genes. Differences in the Log2 gene expression between the functional intervention groups with the control were analysed by unpaired t test. Bivariate Spearman’s correlations (non-parametric) were used to measure the association between the relative fold change in gene expression (2^−ΔΔCt^) and change in cardiometabolic disease risk markers (visit 2-visit 1) relative to baseline, the data for the three functional interventions (probiotics, oats, and apples) were combined. P-value ≤ 0.05 was considered significant.

## Results

In this present analysis, PBMC cDNA samples were only available from 59 of the 61 participants from the CABALA study [one participant each from the control and apples group was excluded due to missing samples]. The mean ± SEM for the age of these participants was 52 ± 2 years, BMI 24.9 ± 0.4 kg/m^2^, and TC 5.10 ± 0.11 mmol/L, with *n* = 15 each in the probiotic, oats and apples groups, and *n* = 14 in the control group. There were no significant differences in baseline (week 0) characteristics (age, and BMI), circulating BAs, lipid profiles, glucose, gut peptides, and inflammatory markers between the functional intervention groups (probiotics, oats, and apples) compared with the control group (Table 1).

**Table 1:** Baseline characteristics of the probiotics, Oats, apples and control groups^1^

Parameters	Control (*n* = 14)	Probiotic (*n* = 15)	Oats (*n* = 15)	Apples (*n* = 15)
**Characteristics**				
Age (years)	50 ± 3	53 ± 3	50 ± 3	54 ± 3
Sex (M/F)	4/10	4/11	3/12	3/12
BMI, kg/m^2^	24.7 ± 0.9	24.2 ± 0.9	25.0 ± 0.9	25.5 ± 0.9
**Bile acids**				
Total BAs, µM	7.00 ± 0.15	7.29 ± 0.14	7.23 ± 0.14	7.36 ± 0.14
Conjugated BAs, µM	6.03 ± 0.18	6.54 ± 0.17	6.43 ± 0.18	6.66 ± 0.18
Unconjugated BAs, µM	6.45 ± 0.17	6.49 ± 0.16	6.46 ± 0.16	6.52 ± 0.16
Primary BAs, µM	6.11 ± 0.20	6.25 ± 0.19	6.27 ± 0.19	6.34 ± 0.19
Secondary BAs, µM	6.42 ± 0.13	6.82 ± 0.13	6.67 ± 0.13	6.84 ± 0.13
Hydrophobic BAs, µM	6.46 ± 0.15	6.80 ± 0.14	6.70 ± 0.14	6.87 ± 0.14
Hydrophilic BAs, µM	6.06 ± 0.18	6.30 ± 0.17	6.25 ± 0.17	6.36 ± 0.17
**Biochemical profile and gut peptides**				
TC, mmol/L	5.23 ± 0.23	5.34 ± 0.22	4.87 ± 0.22	4.87 ± 0.22
HDL-C, mmol/L	1.67 ± 0.10	1.46 ± 0.09	1.58 ± 0.09	1.49 ± 0.09
LDL-C, mmol/L	3.08 ± 0.20	3.37 ± 0.19	2.86 ± 0.19	2.91 ± 0.19
TAG, mmol/L	1.11 ± 0.10	1.03 ± 0.10	0.87 ± 0.10	1.04 ± 0.10
ApoB, g/L	0.93 ± 0.05	1.03 ± 0.05	0.88 ± 0.05	0.91 ± 0.05
Glucose, mmol/L	5.10 ± 0.11	5.11 ± 0.10	5.09 ± 0.10	4.86 ± 0.10
Insulin, pmol/L	32.7 ± 4.8	25.6 ± 4.6	21.1 ± 4.6	27.5 ± 4.6
GIP, ng/ml	35.7 ± 4.6	35.2 ± 4.5	42.4 ± 4.5	39.0 ± 4.5
**Inflammatory markers**				
IL-6, pg/mL	1.61 ± 0.37	1.79 ± 0.36	1.54 ± 0.36	2.50 ± 0.36
IL-18, pg/mL	177 ± 26	218 ± 25	155 ± 25	199 ± 25
CRP, mg/L	0.79 ± 0.21	1.48 ± 0.59	0.95 ± 0.21	2.43 ± 1.08
TNF-α, pg/mL	0.91 ± 0.06	0.91 ± 0.06	0.83 ± 0.06	0.98 ± 0.06

^1^Values are presented as mean ± SEM. At baseline, subject characteristics between each functional interventions group versus control group were examined with an F-Test, using post model fitting manipulation of results. Baseline in fasting outcomes were analysed by linear model with logarithmic scale adjusted for BMI, age, and sex. Data were back-transformed to the original scale for the reader’s convenience. Abbreviations; apo, apolipoprotein; BMI, body mass index; BAs, bile acids; CRP, C-reactive protein; F, female; GIP, glucose dependent insulinotropic polypeptide; HDL-C, high density lipoprotein-cholesterol; IL, interleukin, LDL-C, low density lipoprotein-cholesterol; M, male; TAG, triacylglycerol; TC, total cholesterol; TNF, tumour necrosis factor-alpha

There were no significant differences in the changes in fasting BAs, lipids, glucose, gut peptides, or inflammatory markers following 8 weeks consumption of the probiotic, oats, and apples compared with the control group (Table 2). However, there were a non-significant tendency for a reduction in serum LDL-C (*p* = 0.067) and apoB (*p* = 0.062) after the apples intervention compared with control group (Table 2).

**Table 2:** Change in fasting bile acids and cardiometabolic risk markers after the 8-week intervention periods compared with baseline

Parameters	Control (*n* = 14)	Probiotics (*n* = 15)	Oats (*n* = 15)	Apples (*n* = 15)
**Bile acids**				
Total BAs, µM	1.12 (0.89, 1.42)	1.14 (0.91, 1.43)	1.04 (0.83, 1.31)	1.22 (0.96, 1.54)
Conjugated BAs, µM	1.18 (0.90, 1.54)	0.87 (0.68, 1.13)	1.01 (0.78, 1.31)	1.18 (0.90, 1.53)
Unconjugated BAs, µM	1.07 (0.79, 1.44)	1.55 (1.16, 2.08)	1.05 (0.78, 1.43)	1.39 (1.02, 1.88)
Primary BAs, µM	1.14 (0.80, 1.63)	1.16 (0.82, 1.63)	1.03 (0.72, 1.46)	1.33 (0.93, 1.90)
Secondary BAs, µM	1.08 (0.90, 1.30)	1.09 (0.91, 1.30)	1.05 (0.88, 1.25)	1.13 (0.95, 1.36)
Hydrophobic BAs, µM	1.09 (0.83, 1.43)	1.06 (0.81, 1.37)	0.97 (0.74, 1.27)	1.22 (0.93, 1.60)
Hydrophilic BAs, µM	1.13 (0.88, 1.46)	1.25 (0.98, 1.60)	1.16 (0.90, 1.50)	1.28 (1.00, 1.65)
**Biochemical profile**				
TC, mmol/L	1.03 (0.96, 1.10)	0.98 (0.92, 1.04)	0.98 (0.91, 1.04)	0.96 (0.90, 1.03)
HDL-C, mmol/L	1.01 (0.96, 1.07)	1.01 (0.96, 1.06)	1.02 (0.97, 1.07)	1.00 (0.94, 1.06)
LDL-C, mmol/L	1.05 (0.99, 1.11)	1.02 (0.96, 1.07)	0.98 (0.93, 1.04)	0.96 (0.91, 1.01)
TAG, mmol/L	1.09 (0.96, 1.25)	1.03 (0.91, 1.17)	0.92 (0.81, 1.05)	0.97 (0.85, 1.10)
ApoB, g/L	1.04 (0.98, 1.09)	1.02 (0.97, 1.07)	0.98 (0.93, 1.03)	0.95 (0.91, 1.00)
Glucose, mmol/L	1.02 (1.00, 1.04)	1.02 (1.00, 1.05)	1.01 (0.98, 1.03)	0.99 (0.96, 1.01)
**Gut peptide**				
Insulin, pmol/L	1.14 (0.91, 1.43)	1.15 (0.93, 1.43)	0.97 (0.78, 1.21)	1.00 (0.80, 1.25)
GIP, ng/ml	1.03 (0.79, 1.34)	1.49 (1.15, 1.92)	0.94 (0.72, 1.22)	1.13 (1.03, 1.76)
**Inflammatory markers**				
IL-6, pg/mL	0.99 (0.86, 1.14)	1.03 (0.90, 1.18)	0.87 (0.75, 1.00)	1.08 (0.93, 1.25)
IL-18, pg/mL	1.01 (0.92, 1.11)	1.05 (0.95, 1.15)	1.01 (0.92, 1.11)	1.05 (0.95, 1.15)
CRP, mg/L	0.64 (0.40, 1.03)	1.06 (0.67, 1.66)	0.73 (0.45, 1.16)	1.21 (0.76, 1.93)
TNF- α, pg/mL	1.01 (0.90, 1.12)	1.05 (0.95, 1.17)	0.96 (0.87, 1.07)	1.04 (0.94, 1.16)

Values represent means with 95% CIs estimated with a linear mixed-model, adjusted for BMI, age and sex. Group effect for change over the 8-week intervention period calculated as log week 8– log week 0 (baseline), evaluated by a marginal linear mixed model with post hoc analysis of differences between groups, with Bonferroni adjustment. *P* ≤ 0.05 was deemed significant. Abbreviations; apo, apolipoprotein; BAs, bile acids; CRP, C-reactive protein; GIP, glucose dependent insulinotropic polypeptide; HDL-C, high density lipoprotein-cholesterol; IL, interleukin; LDL-C, low density lipoprotein-cholesterol; TAG, triacylglycerol; TC, total cholesterol; TNF, tumour necrosis factor-alpha

### Peripheral blood monocular cell gene expression

The fold change in PBMC mRNA expression in response to the control, probiotic, oats, and apples interventions relative to the baseline study visit are shown in Table 3. For the *TLR4* inflammatory gene, there was an upregulation in the fold change in mRNA expression in the control group relative to baseline compared with the probiotics and apples groups (Table 3). There was no significant difference in the expression of other genes determined in PBMC after any of the functional interventions compared with the control group.

**Table 3:** Log2 mRNA gene expression following the 8 week interventions relative to baseline

Gene	Control (*n* = 14)	Probiotics (*n* = 15)	Oats (*n* = 15)	Apples (*n* = 15)
**Bile acid synthesis**				
*GPBAR1*	0.10 ± 0.10	0.09 ± 0.07	0.02 ± 0.08	-0.02 ± 0.10
*CYP27A1*	0.09 ± 0.10	0.10 ± 0.13	-0.11 ± 0.17	-0.20 ± 0.10
**Lipid regulation**				
*LDL-R*	0.26 ± 0.10	0.10 ± 0.08	0.03 ± 0.12	0.11 ± 0.11
*HMGCR*	0.16 ± 0.11	-0.01 ± 0.09	0.13 ± 0.08	-0.01 ± 0.07
*SREBF1*	0.11 ± 0.08	0.07 ± 0.08	0.02 ± 0.08	0.02 ± 0.10
*PPARD*	0.27 ± 0.09	0.02 ± 0.09	0.17 ± 0.14	-0.02 ± 0.13
*ACAT1*	0.07 ± 0.11	-0.03 ± 0.09	0.27 ± 0.12	0.09 ± 0.08
**Gastrointestinal hormone**				
*GIPR*	0.18 ± 0.16	-0.07 ± 0.08	0.24 ± 0.16	0.07 ± 0.19
**Inflammation**				
*TLR4*	0.34 ± 0.10	-0.01 ± 0.10*	0.03 ± 0.16	0.03 ± 0.10^†^
*TNFSF14*	0.27 ± 0.14	-0.16 ± 0.20	0.32 ± 0.11	-0.08 ± 0.12
*NR1H3*	0.08 ± 0.07	0.19 ± 0.08	-0.03 ± 0.09	0.03 ± 0.11

Relative changes in mRNA gene expression are presented as mean ± SEM and are log2 transformed. Differences in the relative mRNA gene expression between each of the functional interventions and the control group were analysed by unpaired t test and *p* < 0.05 was considered significant. *p = 0.024, †*p* = 0.042. Abbreviations; *ACAT1*, acetyl-CoA acetyltransferase 1; *CYP27A1*, cytochrome P450 family 27 subfamily A member 1; *GIPR*, glucose-dependent insulinotropic polypeptide receptor; *GPBAR1*, G protein-coupled bile acid receptor 1; *HMGCR*, 3-hydroxy-3-methylglutaryl coenzyme A reductase; *LDL-R*, low-density lipoprotein receptor; *NR1H3*, nuclear receptor subfamily 1 group H member 3; *PPARD*, peroxisome proliferator activated receptor d; *SREBF1*, sterol regulatory element binding transcription factor 1; *TLR4*, toll-like receptor 4; *TNFSF14*, tumour necrosis factor (ligand) superfamily member 14

We examined the correlations between the relative fold change in mRNA gene expression and changes in fasting BAs and cardiometabolic risk markers following 8-week interventions in the functional intervention groups combined (probiotic, oats and apples, *n* = 45). Near significant moderate negative correlations were evident between the fold change in *GPBAR1* mRNA expression and changes in total (*r*=-0.308, *p* = 0.05) and secondary (*r*=-0.316, *p* = 0.05) BAs. Also, there were near significant moderate inverse associations between the fold change in *SREBF1* mRNA gene expression and changes in HDL-C (*r*=-0.330, *p* = 0.05) and glucose (*r*=-0.372, *p* = 0.05). The change in HDL-C concentration in response to the functional interventions was also inversely correlated with the fold change in *HMGCR* mRNA gene expression (*r*=-0.312, *p* = 0.05). Furthermore, there was a negative correlation between the fold change in *GIPR* mRNA gene expression and change in glucose (*r*=-0.320, *p* = 0.05). Additionally, there were positive correlations found between the fold change in *TLR4* (*r* = 0.314, *p* = 0.05) and *TNFSF14* (*r* = 0.327, *p* = 0.05) mRNA gene expression and change in IL-6 and IL-18 concentrations, respectively.

The relative fold change in mRNA gene expression for *CYP27A1*, *LDL-R*, *PPARD*, *NR1H3*, and *ACAT1* after the functional interventions were not found to be correlated with changes in fasting BAs and cardiometabolic risk markers.

## Discussion

In this explorative study performed within the framework of the CABALA study, we observed an upregulation in the fold change in relative mRNA gene expression for *TLR4* after consumption of the control for 8-weeks compared with the probiotic and apple interventions. Due to the limited differences in relative mRNA gene expression between the functional interventions and control, we performed correlational analysis to provide some insights into the potential relationships been changes in the PBMC gene expression and circulating cardiometabolic disease risk markers in the functional intervention groups combined. Moreover, the relationships we found between the fold change in relative mRNA expression for *GPBAR1*, *TLR4*, *TNFSF14* and *SREBF1* and cardiometabolic disease risk markers in the functional groups combined were largely confirmatory of in vitro and animal studies.

In the present study, the 8 week probiotic (*L. reuteri*) and polyphenol-rich apple interventions appeared to have attenuated the increase in PBMC *TLR4* gene expression observed with the control. Interactions between the gut microbiota and toll-like receptors on intestinal epithelial cells and immune cells helps to maintain a balanced immune response and gut homeostasis. TLR4, a transmembrane protein, is an important mediator of the innate immune system and acts as a sensor for microbial components. It mounts an adaptive immune response to bacterial lipopolysaccharide and other pathogens via activation of nuclear factor (NF)-_k_B signalling pathways, promoting the synthesis of pro-inflammatory cytokines [19]. Although the cholesterol-lowering effects of *L.reuteri* (a probiotic with BSH activity) are well recognised [20], studies are emerging which suggest that this probiotic can also have favourable effects on the immune system and intestinal microenvironment (by inhibiting the growth of various pathogens) [21]. This has been demonstrated in newborn rats with necrotizing enterocolitis, in which supplementation with *L. reuteri* strains DSM17938 and ATCC PTA 4659 reduced the incidence and severity of this intestinal disease [21]. Lower intestinal inflammation was attributed to a down-regulation of TLR4 and NF-kB mRNA gene expression and inhibition of the downstream production of pro-inflammatory cytokines [22]. Therapeutic benefits of *L. reuteri* have also been observed in other animal models of chronic gut inflammation, with growing interest as a potential treatment in human inflammatory bowel disease [21].

Probiotic bacteria with BSH activity are also thought to have beneficial effects on the inflammatory response by increasing unconjugated BAs, which allows the gut microbiota to transform these further to secondary BA [2, 13]. Although unconjugated BAs deoxycholic acid (DCA) and lithocholic acid (LCA) are often considered to be detrimental to the intestinal barrier function, studies in mouse models of gut inflammation have reported rectal infusions of DCA/LCA to have anti-inflammatory effects via activation of GPBAR1/TGR5 signalling [20]. Although we have previously reported chronic intake of the probiotic *L.reuteri* to increase postprandial circulating levels of unconjugated and hydrophobic BA compared to control, differences were not evident in the fasting state [13]. Furthermore, the relative PBMC *GPBAR1* mRNA expression was not different between the functional interventions suggesting that findings in animal models may not be as easily translatable to humans.

Supplementation of the diet of grass carp fish with apple polyphenols (AP) for 60-days has also been shown to inhibit TLR4 dimerization in a dose-dependent manner in the intestinal tissue of the fish [23]. This is an essential step for *TLR4* mRNA gene expression activation, with fish consuming 5 and 10 g/day of AP having lower levels of TLR4 compared with the 1 g/day group. The transcription of pro-inflammatory cytokine genes such as IL-6 and TNF-a were down-regulated after the suppression of TLR4 signalling pathway in grass carp [22]. These findings were associated with an enhancement of intestinal antioxidant capacity which the authors proposed could also indirectly contribute to the anti-inflammatory response but also have positive impact on intestinal barrier function. Although we only observed moderate relationships between TLR4 mRNA expression and the changes in fasting IL-6 and IL-18 concentrations, further studies are needed to shed light on the mechanisms underlying the effects of AP on the inflammatory response and gut health.

In addition, in our study, we observed a negative correlation between the change in GPBAR1 mRNA expression in response to the functional interventions and plasma primary and secondary BA. This relationship was unexpected but suggests that circulating plasma BA may negatively regulate the expression of GPBAR1 and/or impact on expression via effects on glycaemia control or inflammatory responses. BAs act as ligands for cell surface G-protein-coupled receptors, such as GPBAR1 which plays an important role in orchestrating postprandial metabolic processes via effects on gut-liver axis signalling, impacting on lipid metabolism, inflammation, and glycaemic control. Activation of GPBAR1 has been associated with a reduction in insulin resistance and inflammatory response, with secondary BA more potent ligands than primary or tertiary BAs [2]. Activation of GPBAR1 has been associated with a reduction in insulin resistance and inflammatory response, with secondary BA more potent ligands than primary or tertiary BAs. However, very little is known about the impact of dietary components on mRNA receptor expression. Furthermore, in the current study there was a negative correlation between the fold change in SREBF1 mRNA gene expression and the change in fasting plasma glucose in the functional interventions group combined. SREBP-1a and SREBP-1c are encoded by SREBF1 and have been linked to type 2 diabetes, glycemia, and insulin resistance in humans [24, 25]. It is known that in the liver, insulin modifies gene expression via transcriptional upregulation and processing of SREBP-1c [26]. This indicates that SREBP-1c not only is responsible for the lipogenic impacts of insulin in the liver but also contributes to the suppression of the gluconeogenesis leading to decrease of glucose production. Also, we found a negative correlation between the fold change in GIPR mRNA gene expression and the change in glucose. GIPR is known to stimulate insulin resistance and cytokine expression in human adipocytes [27]. Previous studies in obese adults and normal mice have shown that consumption of high fat diet could affects GIPR and SREBF1 genes expression signalling that prevent obesity and improve insulin sensitivity [28–30]. However, further studies examining the effects of the functional interventions on SREBF1 and GIPR genes expression and impact on insulin sensitivity are needed in healthy humans.

The strength of this present study is the use of PBMC which circulate in the blood and are exposed to circulating metabolites formed following the chronic functional interventions. Limitations include the small number of subjects in each intervention group, which may have impacted on the power to detect significant differences in the relative mRNA gene expression between the functional intervention groups compared to the control group. Furthermore, we focussed specifically on key genes in PBMC involved in BA, inflammation and lipid metabolism so we cannot discount that other genes or proteins involved in these pathways may have been modulated by the functional interventions compared with the control.

In conclusion, the daily consumption of probiotics and apples for 8 weeks attenuated the upregulation of the PBMC *TLR4* mRNA gene expression observed in response to the control intervention. These observations were largely confirmed by the correlations between the fold change in gene expression (*GPBAR1*, *SREBF1*, *GIPR*, *TLR4* and *TNFSF14*) and change in cardiometabolic disease risk markers after the function interventions.

## Abbreviations

ACAT1: Acetyl-CoA acetyltransferase 1
BSH: Bile salt hydrolase
CYP27A1: Cytochrome P450 family 27 subfamily A member 1
GIPR: Glucose-dependent insulinotropic polypeptide receptor
GPBAR1: G protein-coupled bile acid receptor 1
HMGCR: 3-hydroxy-3-methylglutaryl coenzyme A reductase
LDL-R: Low-density lipoprotein-receptor
NR1H3: Nuclear receptor subfamily 1 group H member 3
PBMC: Peripheral blood mononuclear cells
PPARD: Peroxisome proliferator activated receptor d
SREBF1: Sterol regulatory element binding transcription factor 1
TLR4: Toll-like receptor 4
TNFSF14: Tumour necrosis factor (ligand) superfamily member 14
